# Syndrome of inappropriate antidiuretic hormone in a patient with leptomeningeal carcinomatosis

**DOI:** 10.1186/s13256-016-0862-2

**Published:** 2016-03-31

**Authors:** Roosevelt Boursiquot, Danielle Krol, Sameul Hanif, Javier Rojas, Maneesh Jain

**Affiliations:** 1 Department of Medicine, Drexel University College of Medicine, Philadelphia, PA USA; 2 Department of Pathology, Drexel University College of Medicine, Philadelphia, PA USA; 3 Department of Radiology, Drexel University College of Medicine, Philadelphia, PA USA; 4 Department of Medicine, Division of Hematology/Oncology, Drexel University College of Medicine, and I. Brodsky Associates, Philadelphia, PA USA

**Keywords:** Leptomeningeal carcinomatosis, Esophageal adenocarcinoma, Syndrome of inappropriate antidiuretic hormone

## Abstract

**Background:**

Leptomeningeal carcinomatosis is a condition in which metastatic cancer cells infiltrate the meninges of the brain and spinal cord, progressing to also involve the cerebrospinal fluid. Incidence of leptomeningeal carcinomatosis arising from an esophageal cancer is particularly rare.

**Case presentation:**

Here, we present a case report of a 76-year-old Caucasian man with a history of esophageal adenocarcinoma status-post chemoradiation followed by resection. He was admitted to our unit for intractable headache, nausea without emesis, anorexia, weakness, gait instability, delirium, syncope, and near syncope. Our diagnostic workup revealed leptomeningeal carcinomatosis and syndrome of inappropriate antidiuretic hormone. Our patient was treated with lumbar puncture for the headache, methotrexate for the leptomeningeal carcinomatosis, and table salt for the syndrome of inappropriate antidiuretic hormone. Despite our best efforts, our patient died 6 weeks posttreatment.

**Conclusions:**

Understanding the molecular pathogenesis of the development of syndrome of inappropriate antidiuretic hormone associated with leptomeningeal carcinomatosis from metastatic esophageal adenocarcinoma would help us to identify patients at risk and treat them accordingly.

## Background

The incidence of leptomeningeal carcinomatosis (LC, also termed carcinomatous meningitis or neoplastic meningitis), a rare late complication of tumors [1, 2], is low, occurring in only 5 % of all patients with metastatic solid tumors [3, 4]. However, the prognosis of LC is grim with the survival median at a few weeks to a couple of months [3, 4]. Common symptoms associated with LC include headache, lethargy, seizures, gait disturbances, incontinence, behavior changes, nuchal rigidity, neck pain, and cranial nerve involvement such as impaired vision, diplopia, hearing loss, and vertigo [2, 5]. We report a case involving a 76-year-old Caucasian man with esophageal cancer who had undergone chemoradiation followed by resection and who was recently diagnosed with LC and displayed signs and symptoms of the syndrome of inappropriate antidiuretic hormone (SIADH).

## Case presentation

**Fig. 1:**
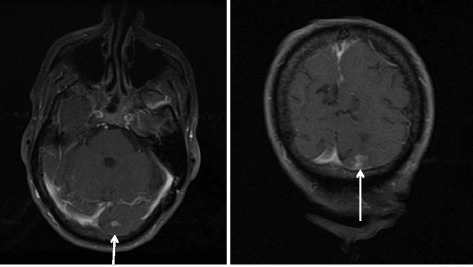
Coronal T1-weighted postcontrast magnetic resonance images demonstrating a subcortical density (*arrows*) in the subcortical region of the inferior left occipital lobe

**Fig. 2:**
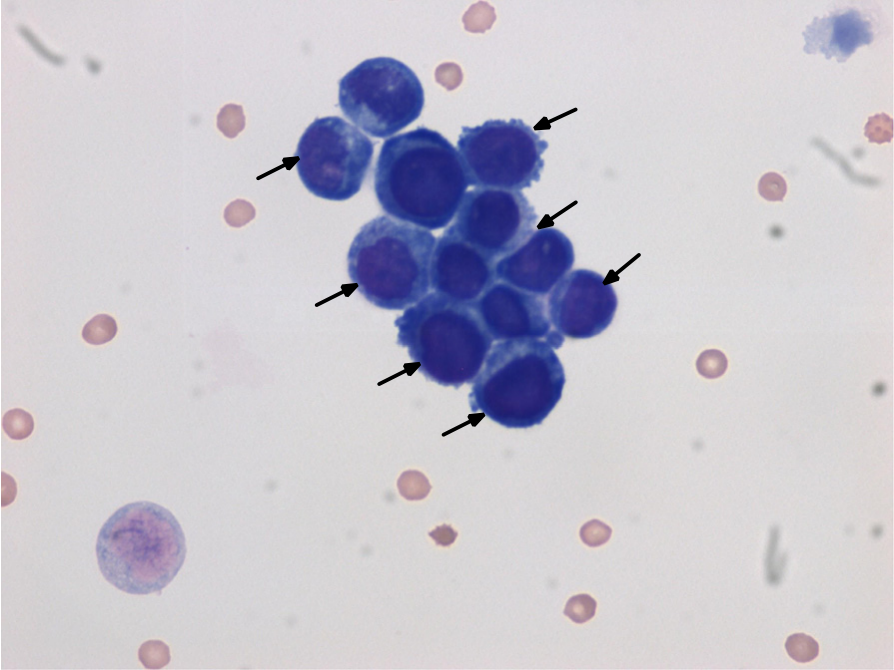
Cytologic analysis of cerebral spinal fluid from a lumbar puncture shows metastatic adenocarcinoma (*arrows*) (Romanowsky stain, magnification ×60)

Our patient had a 30 pack year history of cigarette smoking and a recent history of poorly differentiated adenocarcinoma. Staging was remarkable for yT2N1M0, stage IIB, lower thoracic esophageal adenocarcinoma. Our patient had been treated with chemoradiation followed by minimally invasive esophagectomy. He presented with intractable headache, hypertension (about 200/100 mm Hg), weakness, moderate hearing loss, urinary incontinence, delirium as evidenced by waxing and waning mental status changes, gait disturbance, nausea, and dizziness. We suspected an acute stroke, but a computed tomography (CT) scan of his brain was negative for acute hemorrhage, although it did show evidence of past strokes. Magnetic resonance imaging (MRI) findings showed possible vasculitis or leptomeningeal carcinomatosis (Fig. 1). Results of his four-vessel angiography were unremarkable. Findings were negative for erythrocyte sedimentation rate, antinuclear antibodies, and antineutrophil cytoplasmic antibodies, and thus we ruled out vasculitis. A lumbar puncture initially showed an elevated opening pressure of 30 cm H_2_O but was negative for infection. Cytologic analysis of his cerebrospinal fluid (CSF) was positive for adenocarcinoma (Fig. 2), and leptomeningeal carcinomatosis was diagnosed. A positron emission tomography (PET)/CT scan performed 4 weeks earlier had shown no sign of disease progression. Electroencephalography did not identify seizure or status epilepticus.

Our patient’s leptomeningeal carcinomatosis was complicated by hyponatremia. His plasma sodium level was as low as 123 mmol/L and his serum osmolarity was less than 280 mOs/kg, findings that, in combination with his clinical picture, are consistent with the diagnosis of SIADH (Table 1) [1, 2]. Based on these findings, we concluded that our patient’s hyponatremia was most likely secondary to paraneoplastic SIADH, presumably caused by the leptomeningeal carcinomatosis.

**Table 1:** Patient electrolytes depicting hyponatremia, consistent with a diagnosis of syndrome of inappropriate antidiuretic hormone

Electrolytes	Plasma (reference intervals)	Urine (reference intervals)
Sodium (Na)	123 (135–148 mmol/L)	97 (10–250 mmol/L)
Potassium (K)	3.2 (3.3–5.0 mmol/L)	34 (1.5–100 mmol/L)
Chloride (Cl)	86 (96–110 mmol/L)	122 (20–250 mmol/L)
Urea nitrogen	10 (5–25 mg/dL)	201 (10–1500 mg/dL)
Creatinine (Cr)	0.47 (0.8–1.4 mg/dL)	56 (10–250 mg/dL)
Osmolality	269 (280–305 mOs/kg)	455 (350–1200 mOs/kg)

Our patient’s hyponatremia was managed with salt tablets and his sodium levels eventually returned to normal. His hypertension was controlled with antihypertensive medications. After each of four lumbar puncture sessions, our patient stated that his headaches had resolved. To treat his leptomeningeal carcinomatosis, we started intrathecal methotrexate (MTX) at 12.5 mg in normal saline twice weekly. Our patient underwent an Ommaya reservoir placement by the neurosurgery team as the family wished to continue care with hospice support at home. A few days later, our patient was discharged and advised to continue twice-weekly intrathecal MTX treatments until CSF findings were negative. Unfortunately, our patient died 9 days later, 6 weeks after the diagnosis of leptomeningeal carcinomatosis and 14 months after the diagnosis of esophageal adenocarcinoma. Lukas *et al.* have reported that LC progresses very quickly and based on a systematic review of the literature, survival is within 2.5 to 16 weeks from the time of diagnosis [6], which was the case with our patient.

## Discussion

Leptomeningeal carcinomatosis (LC) is a rare complication of tumors derived from breast, lung, melanoma, and prostate tissue [3]. Cancer cells infiltrate the meninges of the brain and spinal cord and eventually the CSF [4, 5]. Here, we report a case of a 76-year-old Caucasian man with a history of esophageal adenocarcinoma status postchemoradiation followed by resection who presented with signs and symptoms associated with LC. Notably, our patient reported an intractable headache, weakness, moderate hearing loss, urinary incontinence, delirium as evidenced by waxing and waning mental status changes, gait disturbance, nausea and dizziness, all classic clinical presentations for patients with LC. Our patient, however, did not report clinical symptoms such as diplopia, emesis, facial twitch, or seizure. Electroencephalography did not identify seizure or status epilepticus but did show frontal intermittent rhythmic delta activity (FIRDA) bursts, which could have been due to increased intracranial pressure or a deep midline lesion in the brain. The presenting symptoms were corroborated with clinical findings such as a brain MRI scan (Fig. 1) and CSF cytology analysis showing adenocarcinoma (Fig. 2). A recent PET/CT scan and exhaustive tests and imaging studies did not show other primary sources for the adenocarcinoma found in the CSF.

Leptomeningeal carcinomatosis can be a complication of lung cancer. Despite our patient’s smoking history, exhaustive tests and imaging eliminated other primary sources for the adenocarcinoma found in the CSF. In this case, we felt certain of the diagnosis of leptomeningeal carcinomatosis metastasis from a primary esophageal adenocarcinoma. A total of four lumbar punctures were performed on our patient, of which two were for intrathecal methotrexate (MTX) treatment. Following each lumbar puncture, our patient reported significant relief of his headache. The goal of the MTX treatment was to continue until CSF studies revealed negative cytologies.

Our patient’s LC was complicated by hyponatremia with a plasma sodium level as low as 123 mmol/L. The general findings supporting our patient’s SIADH diagnosis included: (a) low plasma sodium and serum osmolarity, (b) inappropriately elevated urine osmolality (above 100 mOs/kg), (c) urine sodium concentration usually above 40 meq/L, (d) low blood urea nitrogen (BUN) and serum uric acid concentration, (e) a relatively normal serum creatinine concentration, (f) normal acid-base and potassium balance, and (g) normal adrenal and thyroid function. Laboratory findings for our patient during his hospital stay met most of the aforementioned SIADH criteria (Table 1), suggesting the etiology was secondary to paraneoplastic SIADH, presumably due to the LC.

## Conclusions

The case report highlights the rarity of esophageal cancer metastasizing to the leptomeninges and inducing SIADH and other clinical symptoms. Understanding the molecular pathogenesis of the development of SIADH associated with leptomeningeal carcinomatosis from metastatic esophageal adenocarcinoma would help us to identify patients at risk and treat them accordingly.

## Consent

Written informed consent was obtained from the patient for publication of this case report and any accompanying images. A copy of the written consent is available for review by the Editor-in-Chief of this journal.

## Abbreviations

CT: computed tomography
CSF: cerebrospinal fluid
LC: leptomeningeal carcinomatosis
MRI: magnetic resonance imaging
MTX: methotrexate
PET: positron emission tomography
SIADH: syndrome of inappropriate antidiuretic hormone
